# Intraspecific trait variation can weaken interspecific trait correlations when assessing the whole‐plant economic spectrum

**DOI:** 10.1002/ece3.3447

**Published:** 2017-09-21

**Authors:** Daniel C. Laughlin, Christopher H. Lusk, Peter J. Bellingham, David F. R. P. Burslem, Angela H. Simpson, Kris R. Kramer‐Walter

**Affiliations:** ^1^ Department of Botany University of Wyoming Laramie WY USA; ^2^ Environmental Research Institute School of Science University of Waikato Hamilton New Zealand; ^3^ Landcare Research Lincoln New Zealand; ^4^ School of Biological Sciences University of Aberdeen Aberdeen UK

**Keywords:** fine root tissue density, leaf economic spectrum, ontogenetic development, relative growth rate, root economic spectrum, wood density, wood economic spectrum

## Abstract

The worldwide plant economic spectrum hypothesis predicts that leaf, stem, and root traits are correlated across vascular plant species because carbon gain depends on leaves being adequately supplied with water and nutrients, and because construction of each organ involves a trade‐off between performance and persistence. Despite its logical and intuitive appeal, this hypothesis has received mixed empirical support. If traits within species diverge in their responses to an environmental gradient, then interspecific trait correlations could be weakened when measured in natural ecosystems. To test this prediction, we measured relative growth rates (RGR) and seven functional traits that have been shown to be related to fluxes of water, nutrients, and carbon across 56 functionally diverse tree species on (1) juveniles in a controlled environment, (2) juveniles in forest understories, and (3) mature trees in forests. Leaf, stem, and fine root traits of juveniles grown in a controlled environment were closely correlated with each other, and with RGR. Remarkably, the seven leaf, stem, and fine root tissue traits spanned a single dimension of variation when measured in the controlled environment. Forest‐grown juveniles expressed lower leaf mass per area, but higher wood and fine root tissue density, than greenhouse‐grown juveniles. Traits and growth rates were decoupled in forest‐grown juveniles and mature trees. Our results indicate that constraints exist on the covariation, not just the variation, among vegetative plant organs; however, divergent responses of traits within species to environmental gradients can mask interspecific trait correlations in natural environments. Correlations among organs and relationships between traits and RGR were strong when plants were compared in a standardized environment. Our results may reconcile the discrepancies seen among studies, by showing that if traits and growth rates of species are compared across varied environments, then the interorgan trait correlations observed in controlled conditions can weaken or disappear.

## 1 INTRODUCTION

Identifying general principles and trade‐offs that underlie the diversity of organism form and function is a central goal of functional ecology because trade‐offs constrain demographic rates and their linkages to ecosystem processes (Díaz et al., 2016; Shipley et al., 2016). An important step toward this goal, in relation to plants, was the recognition of close coordination among a suite of leaf functional traits: All vascular plant species align around a global spectrum from those with expensive, long‐lived leaves that process resources slowly, to those with low‐cost short‐lived leaves that process resources quickly (Reich, Walters, & Ellsworth, 1997; Wright et al., 2004). Because carbon gain depends on leaves being adequately supplied with water and nutrients, and because construction of other plant organs involves trade‐offs between performance and persistence, it has recently been hypothesized that leaf, stem, and fine root traits will all be closely correlated across species (Freschet, Aerts, & Cornelissen, 2012; Freschet, Cornelissen, Van Logtestijn, & Aerts, 2010; Pérez‐Ramos et al., 2012; Reich, 2014; de la Riva et al., 2016). Inefficiencies caused by one organ operating out of sync with the others would presumably be selected against (Reich, 2014). The “whole‐plant economic spectrum” hypothesis makes two key predictions (Reich, 2014). First, leaf, stem, and fine root traits that are related to resource acquisition and transport will be correlated across all vascular plant species, and will span a single dimension of variation. Second, all of these functional traits will also be correlated with relative growth rate (RGR). However, empirical evidence for this “whole‐plant economic spectrum” is mixed.

On the one hand, correlations across leaf, stem, and fine root traits of both herbaceous and woody plants have been shown in several different vegetation types (Freschet et al., 2010; Pérez‐Ramos et al., 2012; Reich et al., 2008; de la Riva et al., 2016). Many studies have also demonstrated correlations between economic traits of leaves and stems (Brodribb & Feild, 2000; Markesteijn, Poorter, Paz, Sack, & Bongers, 2011) and between leaves and fine roots (Craine, Froehle, Tilman, Wedin, & Chapin, 2001; Reich et al., 2008; Tjoelker, Craine, Wedin, Reich, & Tilman, 2005). On the other hand, several other studies have reported weak correlations among leaf, stem, and root traits, or no correlation at all (Baraloto et al., 2010; Fortunel, Fine, & Baraloto, 2012; Jager, Richardson, Bellingham, Clearwater, & Laughlin, 2015; Pietsch et al., 2014; Weemstra et al., 2016; Wright et al., 2007), indicating that interspecific correlations across organs are not consistently observed. Moreover, while substantial volumes of theory and data support relationships between morphological functional traits and RGRs (Hunt & Cornelissen, 1997; Reich, Tjoelker, Walters, Vanderklein, & Buschena, 1998; Shipley, 2006), correlations observed in the field are sometimes weak or nonexistent (Paine et al., 2015; Poorter et al., 2008; Wright et al., 2010). What can explain these discrepancies?

**Figure 1:**
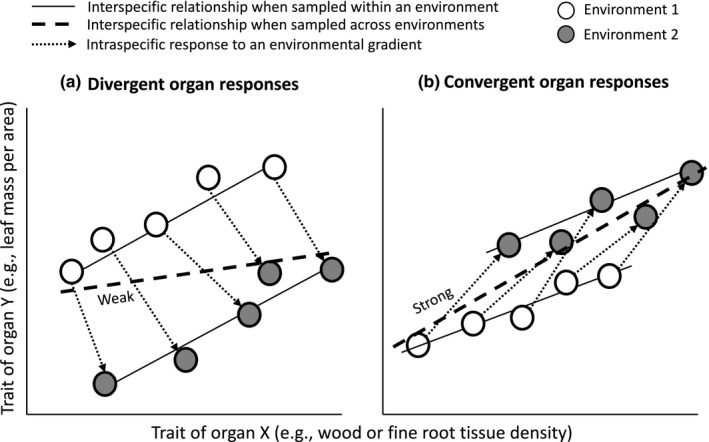
A hypothesis to explain how intraspecific trait variation can decouple the interspecific whole‐plant economic spectrum. (a) If one organ within a species responds positively to an environmental gradient whereas another organ responds negatively, then the strength of the interspecific relationship of traits measured haphazardly from multiple environmental conditions could be weakened or disappear. (b) In contrast, if traits from different organs within species respond in the same direction to an environmental gradient, then the strength of the interspecific relationship of traits measured across environments will remain strong. More generally, if the intraspecific response aligns with the interspecific relationship, then the relationship of traits measured across environments will remain strong. This hypothesis has been extended to multiple organs from the leaf‐based models of Russo and Kitajima (2016)

First, variation in individual‐level access to resources can cause trait correlations within species to differ from the correlations among species (Van Noordwijk & de Jong, 1986). For example, leaf mass per area (LMA) and leaf lifespan are positively correlated among species, reflecting evolutionary adaptation to shade, but intraspecific correlations across light gradients are negative, as ecological acclimation to shade decreases LMA but increases leaf lifespan (Lusk, Reich, Montgomery, Ackerly, & Cavender‐Bares, 2008; Russo & Kitajima, 2016). This could equally apply to traits from different organs: If the two traits show divergent responses to an environmental gradient, then trait correlations among species measured across varied environments could be weakened, or disappear (Figure 1a). In contrast, if traits of different organs respond in the same direction to an environmental gradient, then interspecific comparisons will remain strong (Figure 1b). More generally, if the intraspecific response to environmental gradients aligns with the interspecific relationship, then the correlation of traits measured on species from multiple environments will remain strong. This conceptual hypothesis does not depend on the length of the reaction norms within a species (note that the length of all the dotted lines in Figure 1a,b are approximately equal). Renewed interest in intraspecific trait variation has increased our understanding of multiple processes in ecology (Albert et al., 2010). Intraspecific trait variation is generated by genetic differences among populations, plastic responses to environmental gradients, and ontogenetic changes (Russo & Kitajima, 2016). It remains unclear how intraspecific responses to environmental gradients may affect interspecific trait correlations.

A second potential cause for these discrepancies could be that interspecific differences in ontogenetic development might weaken trait correlations in adults. For example, the LMA of two tree species might be similar at the seedling stage, but then diverge later in development if they differ greatly in final height, because the larger species will undergo a greater increase in LMA (Koch, Sillett, Jennings, & Davis, 2004). Furthermore, models of plant growth predict that the relationship between LMA and growth rate among species will shift from negative in seedlings to nonsignificant or positive in adult plants (Falster, Brännström, Dieckmann, & Westoby, 2011; Gibert, Gray, Westoby, Wright, & Falster, 2016).

We tested the “whole‐plant economics spectrum” hypothesis (Freschet et al., 2010; Reich, 2014) on 56 functionally diverse tree species by evaluating the strength of interspecific trait correlations and correlations between traits and growth rates in juvenile trees in both controlled and field environments, and on mature trees in the field. Expression of plant traits can vary considerably between plants grown in standardized greenhouse conditions versus variable field conditions (Mokany & Ash, 2008; Poorter et al., 2016). We evaluated three hypotheses. First, we predicted that leaf, wood, and fine root traits would be correlated and would each be strong predictors of growth rate among species cultivated in standardized growing conditions (Figure 1a). Second, given that LMA declines (Givnish, 1988) and wood density (WD) can increase (Plavcová & Hacke, 2012) in shaded environments, we predicted that weaker trait correlations would emerge when plants were sampled across lower light gradients in the field (Figure 1a). Third, given that some of the strongest evidence against whole‐plant integration has been found in mature trees sampled in the field (Baraloto et al., 2010), we predicted that mature trees would exhibit weaker interspecific trait correlations than juvenile trees.

## 2 MATERIALS AND METHODS

### 2.1 Growth rate data collection

We selected 56 of the most common tree species from the New Zealand temperate forest flora (see Fig. S1 in Supporting Information), an assemblage that encompasses variation of nearly an order of magnitude in maximum height (6–55 m), >1 order of magnitude in longevity (c. 25 to >700 years), and >1 order of magnitude in leaf lifespan (0.5 to >5 years). Most species are evergreen, except for deciduous *Fuchsia excorticata*, brevi‐deciduous *Sophora microphylla*, and semi‐deciduous *Aristotelia serrata* (McGlone, Dungan, Hall, & Allen, 2004). Phylogenetic relationships among the tree species were extracted from a wider analysis of the phylogeny of vascular plant genera indigenous to New Zealand. In brief, this analysis was based on DNA sequences of the *rbc*L gene supplemented with sequences of the internal transcribed spacer region of the nrDNA repeat or the *mat*K gene for some families (see details in Kramer‐Walter et al., 2016).

We measured traits and growth rates on three types of plants: cultivated juveniles, wild juveniles, and wild mature trees. Table S1 summarizes the attributes of each of the three compiled data sets. For all three types of plants, above‐ground RGR was measured using changes in stem height and diameter over two time points (Baltzer & Thomas, 2007). RGR was calculated as ($\text{ln}\;\left(d_{2}^{2}h_{2}\right)-\text{ln}\left(d_{1}^{2}h_{1}\right))/\left(t_{2}-t_{1}\right)$, where *h*_1_ and *h*_2_ were initial and final stem heights, *d*_1_ and *d*_2_ were initial and final stem diameters, and *t*_1_ and *t*_2_ were initial and final times (Baltzer & Thomas, 2007). These stem dimensions were chosen because *d* ^2^ *h* has been shown to be linearly related to mass (Kohyama, 1991). For consistency, we used the same RGR equation for both juveniles and mature trees. In addition to computing the average RGR for each species, we also computed the 95th percentile of RGR (RGR_95_) of each species as a way to filter out wild plants that were suppressed or unhealthy. RGR_95_ quantifies the observed maximum realized growth rates for a given species in a given site to account for the fact that many individuals will have been experiencing growth suppression through neighborhood competition (Wright et al., 2010). Comparisons of RGR and RGR_95_ within species were virtually identical among cultivated juveniles because these individuals were growing in ideal conditions. However, RGR_95_ was approximately double that of RGR for both wild‐grown juveniles and mature trees.

Cultivated juveniles (seedlings no more than 2 years old) were obtained from native plant nurseries across New Zealand and were grown in the University of Waikato greenhouse in Hamilton (37.7870°S, 175.2793°E). Individual seedlings were grown in separate 8.5‐L pots and were spaced far enough apart so that they did not compete for light. Average light availability in the glasshouse was approximately 20% of full sunlight. Seedlings were grown in a custom‐blend potting medium consisting of a 5:1 mixture ratio of potting mix and propagation sand. The potting mix contained adequate slow‐release fertilizer capsules to ensure that growth was not limited by nutrients, and the sand fraction promoted drainage and simplified root washing. Species were hand‐watered regularly to prevent drought stress. Glasshouse daily average temperature was 15°C. The average duration of growing time across all individuals was 0.31 years (114 days). Between six and 10 individual replicate‐cultivated seedlings per species were used to calculate RGR and RGR_95_.

Growth rates of wild juveniles were estimated using repeated measurements from three sites in New Zealand (Fig. S2). Changes in stem heights and diameters of wild juveniles were measured over periods of between 1 and 3 years. Juvenile trees with negative growth measurements were not included. An average of 47 individuals per species (range: 5–183 individuals per species) were used to calculate RGR and RGR_95_.

Growth rates of mature trees were estimated using repeated tree measurements within the National Vegetation Survey of New Zealand, a national database that contains publicly available vegetation data (https://nvs.landcareresearch.co.nz/). One thousand, four hundred and fifty‐four plots (20 × 20 m) that have been remeasured were used in this study across thirty study sites (Fig. S2). Tree diameters at breast height (dbh) were measured in each time period, but individual tree heights were not. To estimate the height of individual trees, we used species‐specific diameter–height allometric relationships that accounted for the elevation of the site (Holdaway, McNeill, Mason, & Carswell, 2014). Trees with estimated negative diameter growth measurements were not included. The average duration of growing time between measurements was 11.4 years (range: 3–29 years). An average of 977 individuals per species (range: 12–31,398 individuals per species) were used to calculate RGR and RGR_95_.

### 2.2 Trait data collection

We measured LMA, leaf dry matter content (LDMC), and leaf tissue density (LTD) because they are key components of the leaf economic spectrum, which describes variation in fluxes of water, nutrients, and carbon, and these traits reflect the trade‐off between construction cost and longevity (Craine et al., 2001; Poorter, Niinemets, Poorter, Wright, & Villar, 2009; Wright et al., 2004). Species with high LMA, high LTD, and high LDMC are associated with water‐limited, light‐limited, and nutrient‐limited environments (Reich, 2014). Between three and thirty photosynthetic organs (including leaves, leaflets, or phyllodes, hereafter “leaves”) were sampled from each plant, depending on the size per individual leaf. Sample sizes of traits differed across the three datasets: an average of three individuals per species (range: 2–5) for wild‐grown juveniles, nine individuals per species (range: 6–10) for cultivated juveniles, and 62 individuals per species (range: 5–203) for mature trees (Table S1). Mature, fully expanded, healthy leaves (excluding petioles/petiolules) were sampled from canopies of adult trees (using either a shotgun or a telescopic pruner) and from cultivated seedlings. Leaves from wild‐grown seedlings were collected from forest understories that had lower light availability than the greenhouse environment: Average understorey light availability in New Zealand lowland forests has been reported to range from 1.5% to 4.6% of full sun (Coomes et al., 2005; Lusk, Duncan, & Bellingham, 2009), compared with 20% of full sun in the glasshouse. Therefore, leaves of cultivated juveniles are “sun” leaves, whereas leaves of wild juveniles are mostly “shade” leaves (Lusk et al., 2008). Leaf area was measured on a LI‐COR Biosciences LI‐3100C (Lincoln, NE, USA) leaf area meter, leaf thickness was measured using a digital caliper while avoiding midribs on three leaves per individual sample, and leaf fresh mass was measured within 8 hr of sampling after being sealed in plastic bags. Rehydration protocols (Pérez‐Harguindeguy et al., 2013) were trialed, but they did not work well for field‐grown tree leaves because they lost turgor and moisture content overnight; therefore, fresh tissue mass was obtained as soon as possible after sampling for all leaves. Leaves were dried to constant mass at 60°C for at least 48 hr prior to obtaining dry mass. Leaf mass per area was calculated as leaf dry mass divided by fresh leaf area, LDMC was measured as leaf dry mass divided by leaf fresh mass, and LTD was calculated as leaf dry mass divided by leaf fresh volume (i.e., the product of leaf area and thickness).

We measured WD and wood dry matter content (WDMC) because they have been proposed to be key elements of the wood economic spectrum that reflect a fundamental trade‐off between construction cost and longevity (Chave et al., 2009; Freschet et al., 2010). Species with dense stem tissue are associated with water‐limited and nutrient‐limited environments (Reich, 2014). To measure WD on mature trees, one core per tree was collected using a Suunto increment borer with hardened steel bits on trees >10 cm dbh. Cores cannot be extracted from juveniles, so to measure WD on seedlings, a short, straight section (ca. 1–3 cm) of the main stem was cut from the base of the juvenile, and bark was removed by peeling or scraping. The length and orthogonal diameter dimensions of the wood samples were measured using digital calipers, and fresh volume was calculated using the equation for a cylinder. Fresh wood mass was measured within 1 hr of harvesting. Wood dry mass was measured for juvenile stems dried to constant mass at 60°C for at least 48 hr, and for tree cores of adult stems dried to constant mass at 100°C for at least 48 hr. Because of these different WD methodologies between juveniles and mature trees, we do not directly compare traits between juveniles and mature trees. Wood density was calculated as wood dry mass divided by fresh volume, and WDMC was calculated as wood dry mass divided by wood fresh mass.

We measured fine root tissue density and dry matter content (RDMC) because they have been shown to be correlated with fine root respiration rates (Makita et al., 2012), soil resource availability (Poorter & Ryser, 2015; Ryser, 1996), can be coordinated with aboveground traits (Kramer‐Walter et al., 2016), and have been consistently used in other tests of the whole‐plant economic spectrum (Freschet et al., 2010; Pérez‐Ramos et al., 2012; de la Riva et al., 2016). We used the same protocols to measure fine root traits on cultivated juveniles, wild juveniles, and wild mature trees. Subsamples of fine roots, defined as <2 mm diameter, were removed from the roots of each individual and thoroughly cleaned. Roots were identified to species by either tracing the root to an individual tree stem, or using diagnostic morphological traits, such as color, diameter, and presence of nodules (Holdaway, Richardson, Dickie, Peltzer, & Coomes, 2011). The average root diameter across all our samples was 0.5 mm, and the vast majority of the root samples consisted of first‐ through third‐order roots. We avoided suberized root tissue, avoided structural or transport roots, and focused on absorptive roots, but we acknowledge that some transport roots may have been included in some samples. Total root length and root volume were calculated using WinRhizo Pro software (Version 2012b; Regent Instruments Inc., Québec City, Canada) and an Epson Expression 10000XL scanner (Tokyo, Japan). Fresh root mass of each sample was obtained after removing the surface water with paper towels. The root sections were then dried to constant mass at 60°C for at least 48 hr prior to obtaining dry mass. Root tissue density (RTD) was calculated as root dry mass divided by fresh root volume, and RDMC was calculated as root dry mass divided by fresh root mass. Root traits of cultivated juveniles were measured on between six and 10 individual replicates (Table S1). Root traits of wild juveniles were measured on three individual replicates per species found growing in natural conditions on the North Island of New Zealand (Fig. S2). We measured adult fine root traits on at least three individuals per species near Hamilton, New Zealand, and supplemented this with a published root trait dataset of 20 additional species collected at Franz Josef, South Island, New Zealand (Holdaway et al., 2011). All trait measurements followed standardized protocols (Pérez‐Harguindeguy et al., 2013).

Our data spanned most of the global variation in these leaf, wood, and fine root traits. LMA ranged from 28 to 714 g/m^2^, which spans 97% of the global range in LMA (from the 2nd to the 99th percentile) according to the GLOPNET database (Wright et al., 2004). Wood density ranged from 0.25 to 0.82 mg/mm^3^, which spans 85% of the global range in WD (from the 1st to the 86th percentile) according to the dryad database (Chave et al., 2009; Zanne et al., 2009). Fine RTD ranged from 0.07 to 0.54 mg/mm^3^, which spans 74% of the global range in fine RTD (from the 3rd to the 77th percentile) according to a new global synthesis (Freschet et al., 2017).

### 2.3 Data analysis

First, we evaluated the strength and direction of correlations among traits from the three vegetative plant organs. All analyses were conducted at the species‐level using average trait values. Interspecific differences accounted for most trait variation in all cases except for LMA in wild mature trees (Table S2). For all comparisons, we use phylogenetic methods to account for the nonindependence among species due to their shared evolutionary history (Revell, 2009).

We computed an “index of phenotypic integration” that is often used to assess covariance of traits within a population (Cheverud, Wagner, & Dow, 1989) to compare the strength of interspecific multi‐organ trait integration in each of the three groups of plants. Our test of dimensionality included seven functional traits across three organs: LMA, LTD, LDMC, WD, WDMC, fine RTD, and fine root dry matter content (RDMC). These traits are known to be correlated within each organ, so we would not expect a priori the dimensionality to be larger than three in this test, but a dimensionality of two is all that is needed to reject the whole‐plant economic spectrum hypothesis. We performed eigenanalysis on the 7‐dimensional correlation matrix of traits using phylogenetically corrected principal components analysis (PCA) (Revell, 2009, 2012). Phylogenetic PCA results in the calculation of eigenvalues, eigenvectors, component loadings, and scores for each sample unit, and the key property of this method is that the principal component axes are evolutionarily independent (Revell, 2009). The “phenotypic integration index” was computed as the variance of the eigenvalues, $Var\;\left(\lambda \right)=\Sigma_{i=1}^{N}\left({\left(\lambda_{i}-1\right)}^{2}/N\right)$, where *N* is the number of traits, and λ_*i*_ is the eigenvalue from the *i*‐th dimension (Cheverud et al., 1989). Higher values of this index indicate a stronger integration of traits. When traits are uncorrelated, eigenvalues are similar and exhibit low variance. When traits are correlated, the first eigenvalue is much larger than the rest and eigenvalues exhibit high variance. The dimensionality of the trait data is also an indication of the strength of the interspecific whole‐plant economic spectrum. We used Kaiser's rule, where the dimensionality of a dataset is equal to the number of eigenvalues > 1, because one is the average value of an eigenvalue when eigenanalysis is performed on a correlation matrix (Kaiser, 1960). If we observe that only the first eigenvalue is larger than one for a phylogenetic PCA of seven variables, then this will provide strong evidence in support of an interspecific whole‐plant economic spectrum, especially as most trait datasets span multiple dimensions (Laughlin, 2014).

We followed the multivariate analysis with univariate analyses of trait correlations using standard major axis (SMA) regressions within the “smatr” R package (Warton, Duursma, Falster, & Taskinen, 2012). For these univariate analyses, we focus on a single key trait for each organ to illustrate how each organ relates to each other and to growth rates. For these univariate analyses, we used LMA to represent leaves (Wright et al., 2004), WD to represent stems (Chave et al., 2009), and fine RTD to represent roots (Freschet et al., 2010; Pérez‐Ramos et al., 2012; de la Riva et al., 2016). We accounted for phylogenetic relatedness among species using SMA regression on phylogenetically independent contrasts (PICs) for each trait and report both raw trait correlations and PIC correlations. PICs were generated using the phylogeny in Fig. S1 with the pic function in the “ape” R package (Paradis, Claude, & Strimmer, 2004).

Second, we evaluated the strength and direction of correlations between functional traits and both RGR_95_ and RGR. We used two approaches to test this prediction. We accounted for phylogenetic relatedness among species using SMA regression on PICs for individual traits and growth rates, and report both raw correlations and PIC correlations (Tables S3–S5). For this first approach, we computed the average RGR_95_ across all field sites, and the average RGR across all field sites for both wild juveniles and mature trees. To account for the fact that growth rates were measured on wild juveniles and mature trees across a range of different sites that varied in environmental conditions and the number of species per site, we conducted meta‐analyses on the individual site‐level correlations between traits and growth rates. We used the metacor function in the “meta” library of R using *z*‐transformed correlations where site was modeled as a random effect (Schwarzer, 2007). Three sites were used for wild juveniles, and 30 sites were used for wild mature trees (Fig. S2). The number of sites in which each species occurred is reported in Table S6.

Lastly, to test the hypotheses illustrated in Figure 1, we directly compare traits between cultivated and wild‐grown juveniles. Light availability is likely the most prominent difference between the greenhouse (20% of full sun) and forest understories (1.5%–4.6% of full sun) (Coomes et al., 2005; Lusk et al., 2009), although we acknowledge that differences in soil resources (water, nutrients) also played some role (Freschet, Bellingham, Lyver, Bonner, & Wardle, 2013). To determine whether trait rankings are conserved across greenhouse and field‐grown plants, we used SMA regression to evaluate the correlations between cultivated and wild‐grown juvenile traits. To quantify whether the directional differences between juvenile traits in the greenhouse vs. the field differed significantly from zero, we used Wilcoxon signed‐rank tests (a nonparametric alternative to a paired *t* test). To test the hypothesis in Figure 1 that random or haphazard sampling of species across multiple environments could decouple trait correlations compared to those observed within environments, we designed a simulation. For each trait pair, 10,000 datasets were simulated by resampling the empirical data, where the data for each species were randomly drawn from either the greenhouse or the field with equal probability (50% chance of either). Each of the 10,000 datasets contained trait data that were a random mixture of greenhouse and field data for the 56 species. The strength of each pairwise trait correlations was estimated using the *R* ^2^, and the empirically observed *R* ^2^ was compared to the distribution of simulated *R* ^2^. If the observed *R* ^2^ was within the upper 95th quantile of the randomly simulated distribution, this would suggest that haphazard sampling of those trait pairs across multiple environments could weaken interspecific trait correlations. In other words, if trait pairs that exhibit divergent responses within species have lower correlations when sampled across environments, and if trait pairs that exhibit convergent responses within species have the same or stronger correlations when sampled across environments, then this would provide evidence in support of the hypothesis in Figure 1. We do not directly compare wild‐grown juveniles to mature trees to assess ontogenetic effects because interspecific trait correlations between these two types of data could be obscured due to the differences in trait sampling methodology, sampling intensity, and size of plants.

## 3 RESULTS

**Figure 2:**
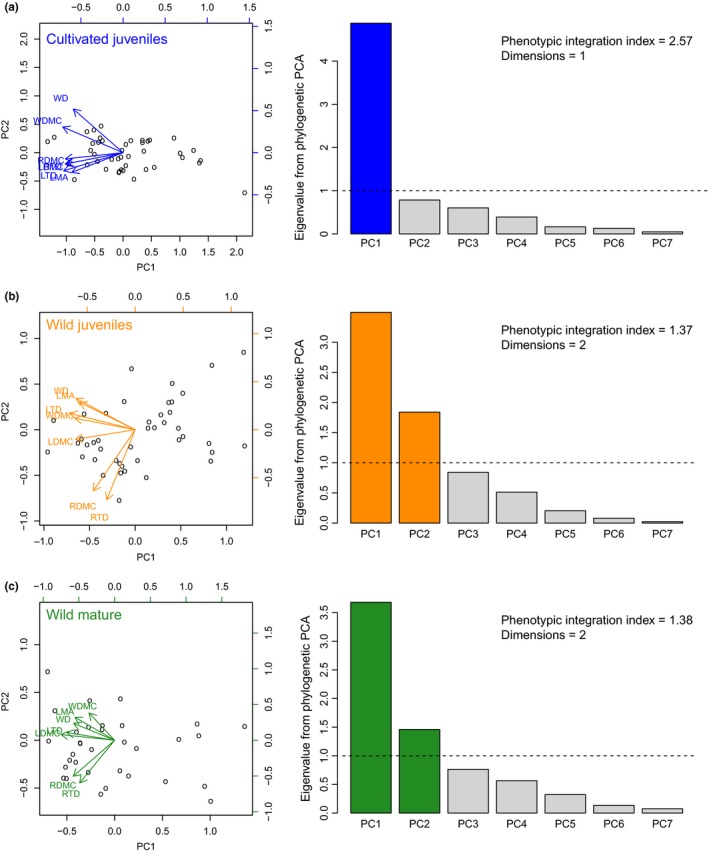
Results of the phylogenetic principal components analysis (PCA) illustrate that cultivated juveniles exhibit the strongest interspecific trait correlations. Left panels illustrate trait loadings on the first two PCA axes, and right panels illustrate eigenvalues associated with each of the seven PCA axes. The index of “phenotypic integration” is computed as the variance of the eigenvalues (Cheverud et al., 1989) and is typically used to assess trait covariance within a population. Dimensionality is estimated using the Kaiser rule (the number of eigenvalues > 1), where each axis with an eigenvalue > 1 exceeds the height of the horizontal dashed line and is shown in color. Seven traits were included in this analysis: leaf mass per area (LMA), leaf tissue density (LTD), leaf dry matter content (LDMC), wood density (WD), wood dry matter content (WDMC), fine root tissue density (RTD), and fine root dry matter content (RDMC). Number of species in each analysis: (a) 43; (b) 43; (c) 33

The whole‐plant economic spectrum hypothesis predicts that leaf, stem, and fine root traits that are related to resource acquisition and transport will be correlated across all vascular plant species and will span a single dimension of variation. This prediction was strongly supported in cultivated juveniles, but not in wild plants (Figure 2). Remarkably, variation in seven functional traits among cultivated juveniles spanned just a single dimension and yielded the highest index of interspecific trait correlations among the three groups tested (Figure 2a). In contrast, traits of wild‐grown juvenile and mature plants spanned at least two dimensions and yielded lower indices of interspecific trait correlations (Figure 2b,c). The increase in dimensionality appears to be driven by a decoupling of fine root traits from both leaf and wood traits (Figure 2b,c). Traits within organs were more strongly correlated than traits among organs in wild juveniles and mature trees (Figure 2b,c, Tables S3–S5).

**Figure 3:**
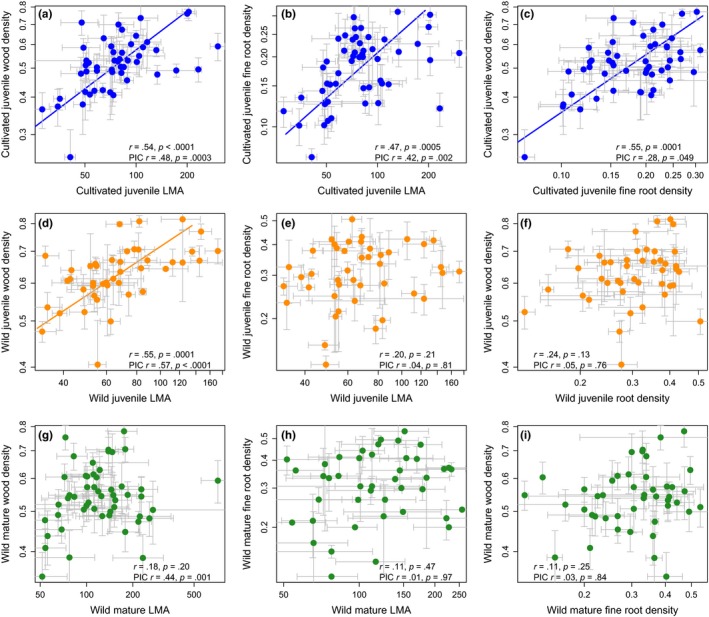
Correlations and significant standardized major axis (SMA) regression lines between cultivated juvenile (a–c), wild juvenile (d–f), and wild mature (g–i) leaf mass per area (LMA, m^2^/g), wood density (mg/mm^3^), and fine root tissue density (mg/mm^3^). The strong coordination of leaf, stem, and fine root tissues in cultivated juveniles is weakened among wild juveniles and wild mature trees. Results of phylogenetically independent contrasts (PICs) are also shown. Lines represent significant SMA regression lines through raw trait data and are only shown if both analyses of raw traits and PICs are significant. Number of species in each regression analysis: (a) 50; (b) 51; (c) 50; (d) 43; (e) 43; (f) 43; (g) 51; (h) 45; and (i) 45. Error bars represent standard deviations

Leaf, wood, and fine root traits of cultivated juveniles were positively correlated across species and across phylogenetic contrasts (Figure 3a–c). Among wild juveniles, LMA and WD were positively correlated (Figure 3d), but there was no correlation between fine root density and either LMA or WD (Figure 3e,f). Although cross‐species correlations showed no significant relationships among leaf, stem, and fine root traits of mature trees (Figure 3g–i), independent contrasts revealed a positive correlation between LMA and WD, indicating that this correlation exists within, rather than among, clades in mature trees (Figure 3g).

**Figure 4:**
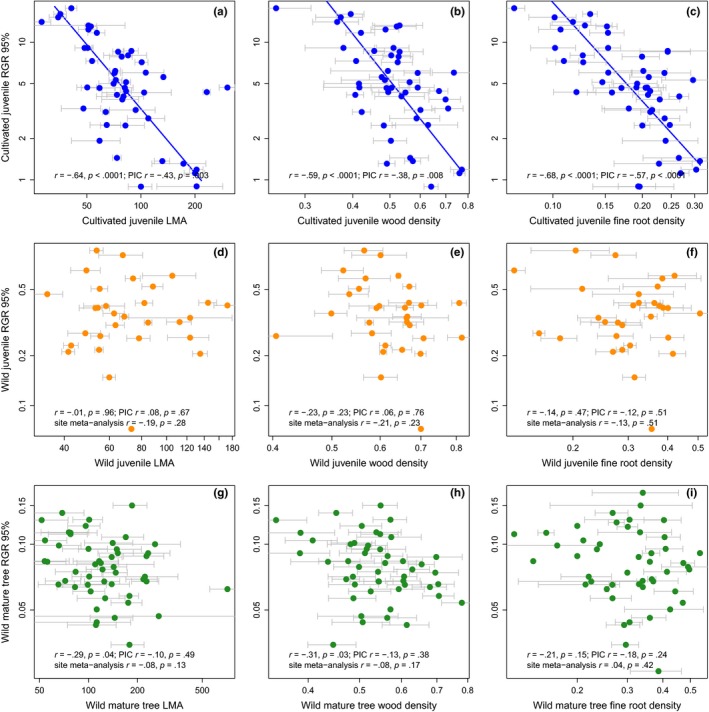
Correlations between the 95th percentile of relative growth rate (RGR_95_) and leaf mass per area (LMA; mg/mm^2^), wood tissue density (mg/mm^3^), and fine root tissue density tissue density (mg/mm^3^) in cultivated juvenile trees (a–c), wild juvenile trees (d–f), and wild mature trees (g–i). The dots in panels (d) through (i) represent the average trait value across sites for each species. Cultivated juvenile leaf, stem, and fine root traits were significantly correlated with growth rates, but traits of wild‐grown trees were decoupled from growth rates. Lines represent significant SMA regression lines through raw trait data and are only shown if both analyses of raw traits and PICs are significant. Number of species in each regression analysis: (a) 47; (b) 46; (c) 47; (d through e) 30 across all sites; 9, 5, and 24 within each of the three sites respectively; (g and h) 49 species across all sites; 8, 6, 10, 17, 3, 22, 14, 12, 17, 12, 17, 15, 18, 27, 14, 12, 16, 13, 28, 23, 10, 24, 5, 12, 32, 11, 15, 26, 15, 22 across each of the 30 sites, respectively; and (i) 47 species across all sites; 8, 4, 9, 15, 3, 19, 11, 10, 15, 12, 16, 14, 17, 25, 14, 11, 14, 12, 28, 21, 10, 22, 5, 12, 27, 10, 14, 23, 14, 21 across each of the 30 sites, respectively. Error bars represent standard deviations of traits among individual plants in (a–c), but they represent standard deviations among sites in (d–i). RGR_95_ is a percentile so we do not plot standard deviations on the *y*‐axis

The whole‐plant economic spectrum hypothesis predicts that leaf, stem, and fine root traits will correlate with inherent variation in RGR. This prediction was strongly supported in cultivated juveniles, but less so in wild plants (Figure 4). Both cross‐species analyses and independent contrasts showed that LMA, WD, and fine RTD of cultivated juveniles were each negatively correlated with above‐ground RGR_95_ (Figure 4a–c). However, meta‐analysis of site‐level correlations found that traits of wild juveniles and mature trees were consistently uncorrelated with growth rates (Figure 4d–i). Site‐level correlations and full details of the meta‐analyses are reported in Tables S7 and S8. When species‐specific RGR_95_ of mature trees was averaged across all sites, they were weakly negatively correlated with both LMA and WD, but these correlations were not supported using phylogenetic contrasts (Figure 4g,h). Analyses of RGR_95_ and RGR were qualitatively similar (Fig. S3 and Figure 3, respectively); the only difference is that the LMA–RGR relationship was nonsignificant for mature trees (Fig. S3).

**Figure 5:**
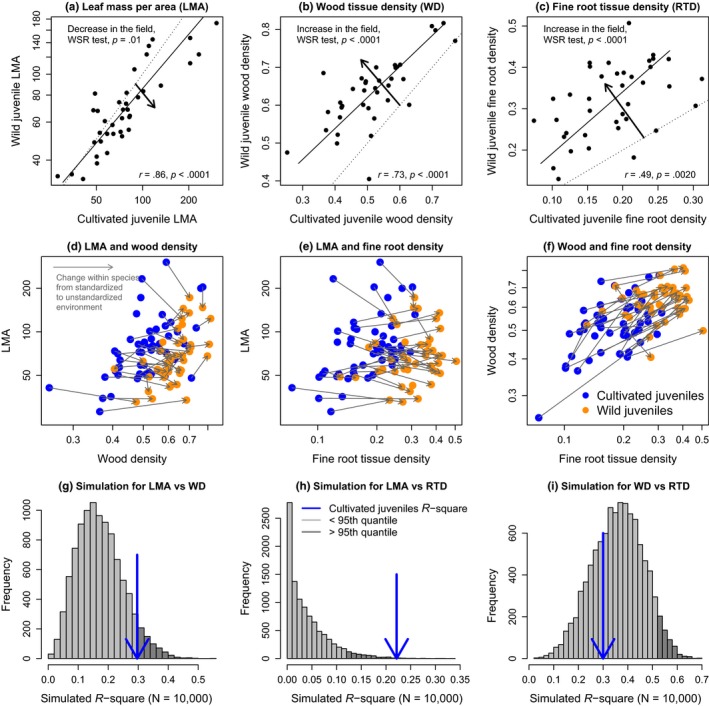
Comparison of traits from cultivated juveniles and wild juveniles. (a) Leaf mass per area (LMA, mg/mm^2^) was lower in shade leaves of wild juveniles compared to sun leaves of cultivated juveniles, whereas (b) wood tissue density (WD, mg/mm^3^) and (c) fine root tissue density (RD, mg/mm^3^) were higher in wild juveniles. The dotted line represents the 1:1 line, and solid lines indicate significant SMA regression lines. SMA regression results are shown in lower right corners, and Wilcoxon signed‐rank (WSR) test results are shown in upper left corners. Number of species in each analysis: (a) 38; (b) 37; (c) 38. The second row of plots (d–f) illustrates directional changes in trait values within juveniles of species from standardized greenhouse environments to unstandardized field environments. The third row of plots (g–i) illustrates the distribution of *R* ^2^ from bivariate trait relationships across the 10,000 simulations, where samples were drawn from the real data by randomly drawing trait data for each species from either the greenhouse or the field. Blue arrows indicate the observed *R* ^2^ for the cultivated juveniles grown in standardized conditions. The dark shaded columns in the histograms indicate the 95th to 100th quantiles

Leaf mass per area (Figure 5a), WD (Figure 5b), and fine root density (Figure 5c) were all positively correlated between cultivated and wild juveniles, indicating that trait rankings are generally preserved between greenhouse and field conditions. However, field‐grown juveniles that were growing in shaded forest understories had lower LMA than cultivated juveniles grown in high light (Wilcoxon signed‐rank [WSR], *p *=* *.01, Figure 5a), whereas field‐grown juveniles had higher wood and fine root tissue densities than cultivated juveniles (Figure 5b,c, WSR test, both *p *<* *.0001), indicating that changes in LMA diverged from the changes in both wood density and root tissue density.

The directional changes of juvenile traits within species from standardized greenhouse to unstandardized field environments are illustrated in Figure 5d–f. For each trait pair, 10,000 simulations were obtained where trait values of each species were drawn from either the greenhouse or the field. For the pairs of traits that exhibited divergent changes within species (LMA vs. WD in Figure 5g; and LMA vs. fine root density in Figure 5h), the simulated correlations were more often much lower than the empirically observed correlations measured in the standardized greenhouse environment. Note that the blue arrows, which indicate the observed correlations in the standardized greenhouse environment, occur at or above the 95th quantile (darker shaded region) of the distribution of simulated correlations (Figure 5g,h). By contrast, the simulated correlations were often strengthened for the pair of traits that exhibited convergent changes within species (wood density vs. fine root density in Figure 5i); in this case, the observed correlation (blue arrow) in Figure 5i is well below the 95th quantile of the distribution of simulated correlations. The divergent changes between LMA and both wood and fine root density could lead to weakened correlations between traits if species are haphazardly sampled from a variety of environmental conditions.

## 4 DISCUSSION

Our results demonstrate that woody plants can express closely correlated traits across vegetative organs but that the whole‐plant economic spectrum can be decoupled in natural ecosystems. We interpret these results to indicate that natural selection has not acted on the variation in leaves, stems, and fine roots in isolation, but has also acted on the covariation among these vital vegetative plant organs, which function in tandem to coordinate fluxes of carbon, water, and nutrients. Individuals that lack the ability to coordinate trait expression among leaves, stems, and fine roots are presumably selected against because of the inefficiencies that arise when organs are not functioning in tandem (Reich, 2014). These results provide support for our three hypotheses: (1) correlations among organs and between traits and RGR are most clearly apparent when plants are compared in standardized environments, (2) divergent responses of traits within species to environmental gradients can lead to weakened trait correlations among species, and (3) mature trees exhibit weak interspecific trait correlations.

Intraspecific trait variation is generated by genetic differences among populations, plastic responses to environmental gradients, and ontogenetic changes (Russo & Kitajima, 2016). The design of this study does not permit us to clearly differentiate between these sources of intraspecific trait variation. Genetic differences among populations and local adaptation may have contributed to some of these patterns. However, plastic trait variation in response to heterogeneous environments in natural ecosystems is the most parsimonious explanation for how intraspecific trait variation influenced the decoupling of the interspecific whole‐plant economic spectrum in our study. The differences in LMA between glasshouse‐ and forest‐grown juveniles (Figure 5) are consistent with well‐known plastic responses to light availability (Givnish, 1988). The differences in WD we observed are also consistent with a recent analysis of xylem plasticity in response to shade (Plavcová & Hacke, 2012). If we were to interpret these results using a genetic explanation, then the seedlings in the field experiments would have had to have been consistently biased toward “low‐LMA” provenances, and the plants in the greenhouse experiments would have had to have been biased toward “high‐LMA” provenances. The odds of this happening consistently across so many species are low. Given that our results agree with well‐established theoretical and empirical responses to light (Givnish, 1988; Lusk et al., 2008), we use the principle of Occam's razor to conclude that phenotypic response to environmental variation is the most parsimonious explanation of the patterns in our data.

If decoupled phenotypes are selected against (Reich, 2014), and if whole‐plant coordination is most clearly expressed in standardized growing conditions, then how has whole‐plant coordination evolved in terrestrial environments where heterogeneity of resources and conditions is ubiquitous? We propose that trait coordination across species is strongest within the same environment, but that these relationships may differ across environments. For example, shade reduces LMA but increases leaf lifespan within species, despite the positive correlation in these traits across species (Lusk et al., 2008; Poorter et al., 2009; Russo & Kitajima, 2016). Similarly, we observed divergent responses among organs to differences in environmental conditions between greenhouse and forest understories, which is (among other things) a gradient in light availability. Our results provide evidence that divergent responses of organs within species across environments can affect the strength of whole‐plant coordination across species (Figure 1a). Trait expression and biomass allocation are known to differ among organs in response to light and nutrient availability (Farrior et al., 2013; Freschet, Swart, & Cornelissen, 2015). In our study, field‐grown juveniles exhibited lower LMA and higher WD, in agreement with other studies (Plavcová & Hacke, 2012; Russo & Kitajima, 2016).

The divergent responses between LMA and WD and between LMA and fine root density conform to the hypothesis illustrated in Figure 1a, whereas the convergent responses between wood density and fine root density conform to Figure 1b. Correlations between divergent traits were weakened in the simulations that compared species sampled randomly across environments (Figure 5f,g), but correlations between convergent traits were strengthened (Figure 5i). These results suggest that if traits respond differently to environmental gradients within species, then correlations between traits among species may weaken or disappear. For example, random or haphazard sampling of juveniles in the field will tend to flatten out species differences in LMA, but heighten differences in WD, as shade‐tolerant species on average occur in darker microsites than light demanders (Lusk et al., 2008). This may also help to explain the recent observation that traits are weak predictors of seedling growth rates at a global scale when traits are acquired from multiple sources and the range of environmental conditions is broad (Paine et al., 2015). If phenotypic coordination is strongest within an environment, then phenotypes that are compared across a range of environments will exhibit decoupled traits and weak relationships between traits and growth rates.

Wide intraspecific variation in RGR of wild juveniles and adult trees (Table S2) indicates that environmental conditions can exert stronger control over growth rates than either traits or species identity, as seen elsewhere (Clark, 2010; Russo et al., 2010). The weak within‐site relationships revealed by meta‐analysis reflect confounding of inherent species differences by variable responses to local environmental gradients. The high intraspecific variation in growth rates among wild juveniles and mature trees is likely driven by site‐level climatic differences and within‐site variation in light availability and soil properties (John et al., 2007; Nicotra, Chazdon, & Iriarte, 1999).

Our results support recent model predictions that LMA will not be negatively correlated with growth rates of mature trees because the cost of building sapwood increases disproportionately with increasing plant size, thereby decreasing the benefit of cheap leaf construction (Falster et al., 2011; Gibert et al., 2016). Surprisingly, our data do not strongly support the predictions and observations that WD will be correlated with growth rates of both juveniles and mature trees (Falster et al., 2011; Gibert et al., 2016; Poorter et al., 2008; Wright et al., 2010). We detected a weak negative correlation between WD and growth rate among mature tree species, but the correlation disappeared in the PICs, suggesting that the generality of this relationship occurs across, rather than within, clades. To complement previous field studies (Poorter et al., 2008; Wright et al., 2010), future studies could clarify the potential role of ontogenetic effects by comparing cultivated juveniles with mature trees grown in standard environments. This task will pose significant challenges, but arboreta and monocultures of trees grown in biodiversity–ecosystem function experiments probably offer the best chance of comparing mature trees of different species grown under conditions that approach the level of standardization achieved in common‐garden experiments.

Our results help to reconcile discrepancies among studies that have evaluated correlations of traits between organs. Studies that have reported strong trait coordination tend to have measured traits on either tree seedlings cultivated in greenhouses (Reich et al., 1998) or on young tissues (twigs < 3 mm diameter) where secondary thickening of xylem has not fully developed (Freschet et al., 2010, 2012). Studies that have not shown trait coordination have mostly measured traits on mature plants growing in varied conditions in the field (Baraloto et al., 2010; Fortunel et al., 2012; Jager et al., 2015). Similarly, studies that have shown strong relationships between traits and growth rates tend to have been conducted under standardized conditions (Hunt & Cornelissen, 1997; Reich et al., 1998), but studies that span broad environmental conditions have reported weak relationships between traits and growth rates (Paine et al., 2015). This dichotomy matches our findings of much clearer expression of whole‐plant integration in controlled environments. It also suggests that if traits are extracted from databases collected from disparate sources or traits and growth rates are compared across a range of unmeasured environmental conditions, then one cannot rigorously evaluate the existence of interspecific whole‐plant integration because of the confounding effects of intraspecific trait variation (Cordlandwehr et al., 2013; Kattge et al., 2011). These results are an important reminder that comparative studies must control for environmental and ontogenetic effects when comparing plant phenotypes, and that traits measured under standardized conditions remain the gold standard for understanding genetically based functional differences among species (Grime et al., 1997). Traits measured on wild plants are clearly valuable for addressing many other research questions, such as identifying broad biogeographic relationships between traits and the environment (Simpson, Richardson, & Laughlin, 2016; Violle, Reich, Pacala, Enquist, & Kattge, 2014), the sources of intraspecific trait variability (Siefert et al., 2015), and the effects of traits on ecosystem processes (Freschet et al., 2012). We recommend that future global tests of the interspecific whole‐plant economic spectrum account for the potentially confounding effects of intraspecific trait variation in response to environmental gradients.

## CONFLICT OF INTEREST

None declared.

## AUTHOR CONTRIBUTIONS

DCL analyzed the data and wrote the paper in collaboration with CHL; DCL, KRK‐W, AHS, CHL, DFRPB, and PJB collected and processed the data and edited the manuscript.

## DATA ACCESSIBILITY

All data used in this study, including all plant traits, by species, for cultivated juveniles, wild juveniles, and wild mature trees are available online at Landcare Research Datastore, https://doi.org/10.7931/j2qn64wd (Laughlin et al., 2017).
