# Supplementary material for: Intraspecific trait variation can weaken interspecific trait correlations when assessing the whole‐plant economic spectrum
Source: Ecol Evol. 2017 Sep 21;7(21):8936–49. doi: 10.1002/ece3.3447 (PMC5677476; doi:10.1002/ece3.3447)
Supplement: Supplementary file 1 [file ECE3-7-8936-s001.docx]

**Supporting Information**

Intraspecific trait variation can decouple the whole-plant economic spectrum

Daniel C. Laughlin, Christopher H. Lusk, Peter J. Bellingham, David F.R.P. Burslem, Angela H. Simpson, Kris R. Kramer-Walter

**Figure S1.** Phylogenetic relationships and illustration of standardized leaf, wood, and stem trait values for each of the 56 native New Zealand tree species.

**Figure S2.** Locations of survey data from throughout New Zealand used in the analysis.

**Figure S3.** Relationships between average RGR and leaf, stem, root traits.

**Table S1.** Summary of data set attributes.

**Table S2**. Percentages of trait and relative growth rate variation explained by interspecific differences in each of the three groups of plants.

**Table S3.** Raw trait and PIC correlations for cultivated juveniles.

**Table S4.** Raw trait and PIC correlations for wild juveniles.

**Table S5.** Raw trait and PIC correlations for mature trees.

**Table S6**. Distribution of the 56 species across the field sites where relative growth rates were obtained for wild-grown mature trees and wild-grown juveniles.

**Table S7.** Results of the meta-analysis of individual site-level correlations between traits and relative growth rates measured on wild juvenile trees.

**Table S8.** Results of the meta-analysis of individual site-level correlations between traits and relative growth rates measured on wild mature trees.


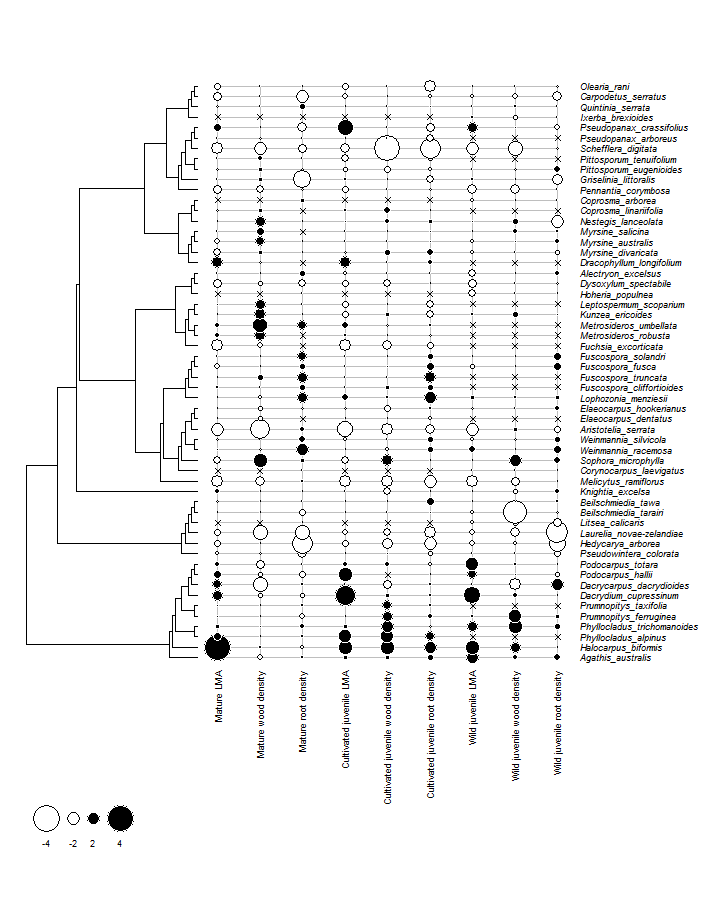


**Fig. S1.** Phylogenetic relationships and illustration of leaf, wood, and stem trait values for each of the 56 native New Zealand tree species. The sizes of symbols are proportional to the trait values standardized to unit variance. Phylogeny from Kramer-Walter et al. ([2016](#_ENREF_1)).


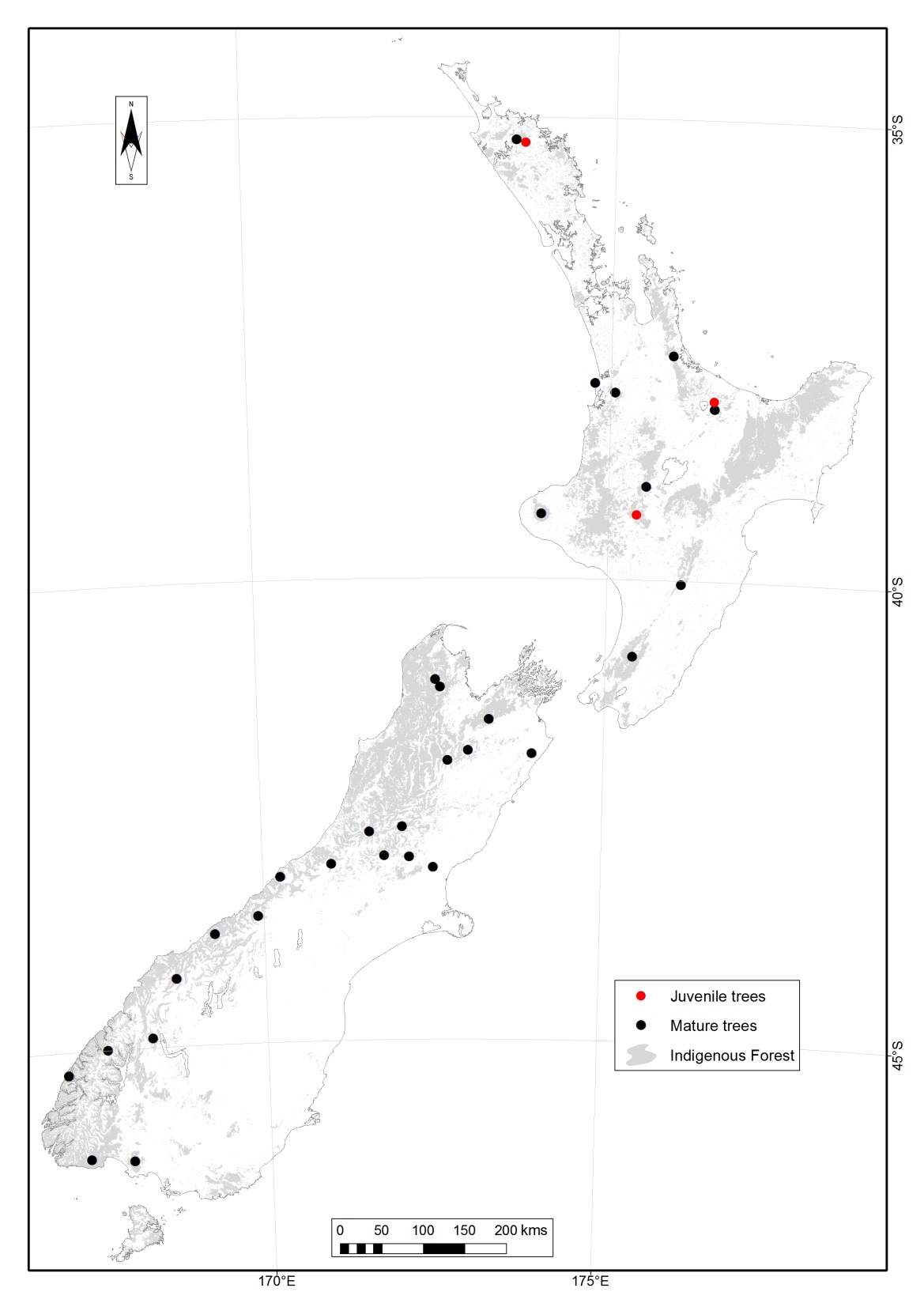


**Fig. S2.** Locations of survey data from throughout New Zealand used in the analysis.


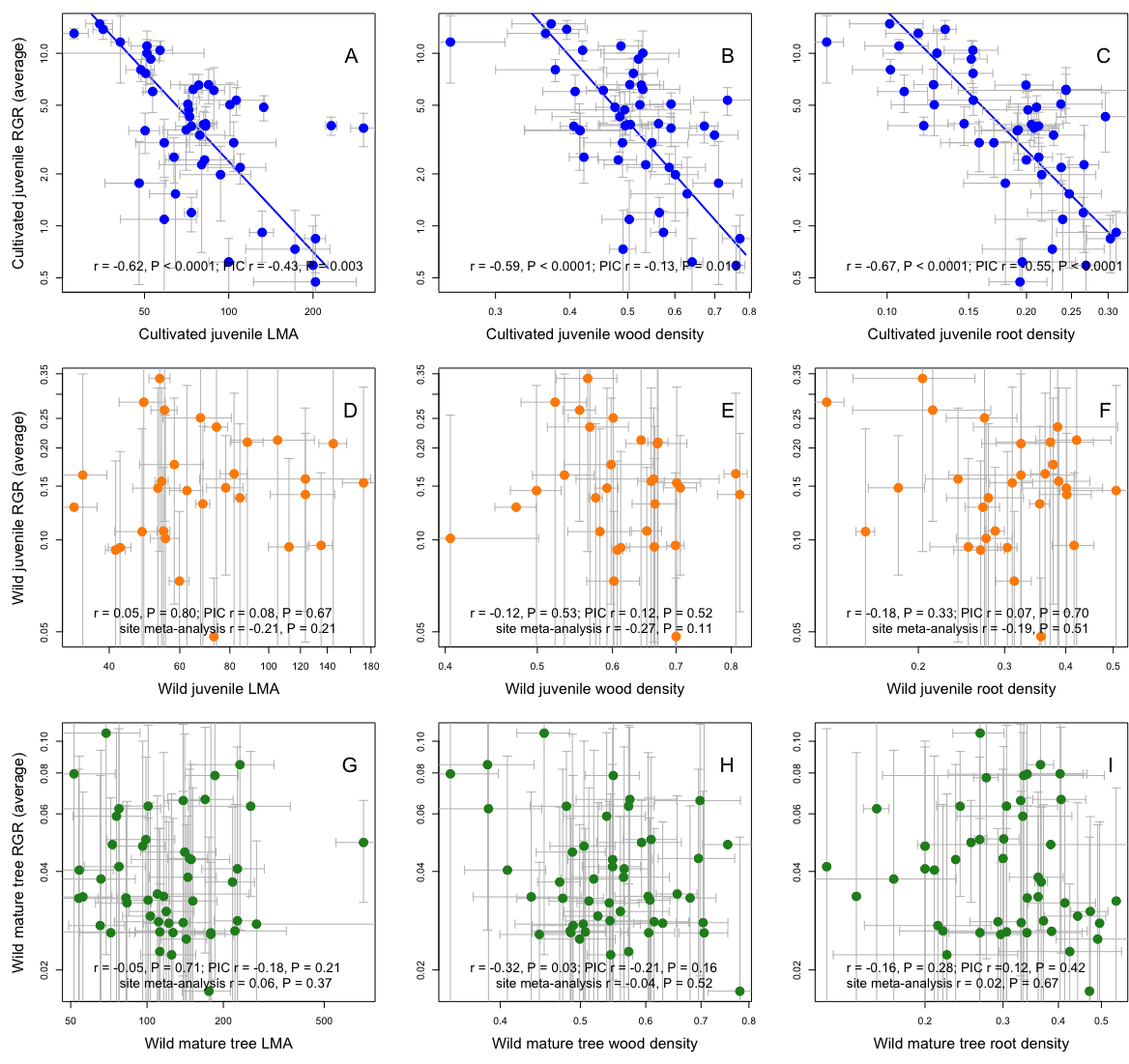


**Fig. S3.** Correlations between the average relative growth rate (RGR) and leaf mass per area (LMA; mg mm^-2^), wood tissue density (mg mm^-3^), and root tissue density tissue density (mg mm^-3^) in cultivated juvenile trees (A–C), wild juvenile trees (D–F), and wild mature trees (G–I). Cultivated juvenile leaf, stem and root traits drive growth rates, but traits on wild-grown trees were decoupled from growth rates. This differs from Fig. 4 in the main text by the analysis of average RGR (rather than RGR_95_). The dots in panels D through I represent the average trait value across sites for each species. Lines represent significant SMA regression lines through raw trait data and are only shown if both analyses of raw traits and PICs are significant. Number of species in each regression analysis: A) 47; B) 46; C) 47; D through E) 30 across all sites; 9, 5, and 24 within each of the three sites respectively; G and H) 49 species across all sites; 8, 6, 10, 17, 3, 22, 14, 12, 17, 12, 17, 15, 18, 27, 14, 12, 16, 13, 28, 23, 10, 24, 5, 12, 32, 11, 15, 26, 15, 22 across each of the 30 sites, respectively; and I) 47 species across all sites; 8, 4, 9, 15, 3, 19, 11, 10, 15, 12, 16, 14, 17, 25, 14, 11, 14, 12, 28, 21, 10, 22, 5, 12, 27, 10, 14, 23, 14, 21 across each of the 30 sites, respectively. Error bars represent standard deviations of traits among individual plants in A-C, but they represent standard deviations among sites in D-I.

**Table S1.** Summary of data set attributes. Given the multiple sources of data, we could not analyse the data at the level of the individual plant. All analyses were done at the level of average traits and growth rates per species.

|  | **Cultivated juvenile tree seedlings** | **Wild juvenile tree seedlings** | **Wild mature trees** |
| --- | --- | --- | --- |
| Size | < 1 m height | < 1 m height | > 10 cm dbh |
| Average duration of growth | 0.31 yrs (114 d) | 2 yrs | 11.4 yrs |
| Average sample size of growth rates per species | 9 (range: 6–10) | 47 (range: 5–183) | 977 (range: 12–31,398**)** |
| Average sample size of trait data per species | 9 (range: 6–10) | 3 (range: 2–5) | 62 (range: 5–203) |
| Environmental conditions | Standardized high resource conditions: high light, water, nutrients | Variable conditions of locations where seedlings were found growing naturally | Variable conditions of locations where trees were found growing naturally |
|  |  |  |  |

**Table S2**. Percentages of trait and relative growth rate variation explained by interspecific differences in each of the three groups of plants. The remaining proportion of variation represents the combination of intraspecific variation plus residual error.

|  | Cultivated juvenile trees | Wild juvenile trees | Wild mature trees |
| --- | --- | --- | --- |
| Leaf mass per area | 56 | 91 | 33 |
| Wood density | 75 | 88 | 70 |
| Root density | 70 | 74 | 65 |
| Relative growth rate | 89 | 18 | 5 |

**Table S3.** Raw trait and PIC correlations for cultivated juveniles. Lower triangle reports raw trait correlations and the upper triangle reports the PIC correlations. Seven traits were included in this analysis: leaf mass per area (LMA), leaf tissue density (LTD), leaf dry matter content (LDMC), wood density (WD), wood dry matter content (WDMC), fine root tissue density (RTD), and fine root dry matter content (RDMC).

|  | LMA | WD | RTD | LDMC | WDMC | RDMC | LTD |
| --- | --- | --- | --- | --- | --- | --- | --- |
| LMA | 1 | 0.41636 | 0.559862 | 0.531735 | 0.604452 | 0.521129 | 0.740967 |
| WD | 0.544692 | 1 | 0.487213 | 0.47676 | 0.896268 | 0.536 | 0.502348 |
| RTD | 0.470634 | 0.547651 | 1 | 0.644915 | 0.626823 | 0.851551 | 0.647855 |
| LDMC | 0.532544 | 0.60507 | 0.692097 | 1 | 0.67461 | 0.653736 | 0.828603 |
| WDMC | 0.6063 | 0.930803 | 0.682113 | 0.702628 | 1 | 0.702849 | 0.665834 |
| RDMC | 0.501126 | 0.599884 | 0.907985 | 0.717303 | 0.733388 | 1 | 0.679291 |
| LTD | 0.711601 | 0.532232 | 0.742687 | 0.874014 | 0.657904 | 0.728519 | 1 |

**Table S4.** Raw trait and PIC correlations for wild juveniles. Lower triangle reports raw trait correlations and the upper triangle reports the PIC correlations. Seven traits were included in this analysis: leaf mass per area (LMA), leaf tissue density (LTD), leaf dry matter content (LDMC), wood density (WD), wood dry matter content (WDMC), fine root tissue density (RTD), and fine root dry matter content (RDMC).

|  | LMA | WD | RTD | LDMC | WDMC | RDMC | LTD |
| --- | --- | --- | --- | --- | --- | --- | --- |
| LMA | 1 | 0.586704 | -0.09891 | 0.402991 | 0.445106 | 0.171331 | 0.721584 |
| WD | 0.553995 | 1 | -0.09761 | 0.375739 | 0.824264 | 0.066329 | 0.62755 |
| RTD | 0.195766 | 0.23683 | 1 | 0.376131 | 0.172913 | 0.918187 | 0.091405 |
| LDMC | 0.583586 | 0.500347 | 0.498141 | 1 | 0.521797 | 0.444119 | 0.683185 |
| WDMC | 0.618808 | 0.838965 | 0.348097 | 0.630716 | 1 | 0.206084 | 0.490526 |
| RDMC | 0.430833 | 0.370649 | 0.940929 | 0.622196 | 0.480504 | 1 | 0.308431 |
| LTD | 0.675187 | 0.498188 | 0.435691 | 0.853589 | 0.59765 | 0.600811 | 1 |

**Table S5.** Raw trait and PIC correlations for mature trees. Lower triangle reports raw trait correlations and the upper triangle reports the PIC correlations. Seven traits were included in this analysis: leaf mass per area (LMA), leaf tissue density (LTD), leaf dry matter content (LDMC), wood density (WD), wood dry matter content (WDMC), fine root tissue density (RTD), and fine root dry matter content (RDMC).

|  | LMA | WD | RTD | LDMC | WDMC | RDMC | LTD |
| --- | --- | --- | --- | --- | --- | --- | --- |
| LMA | 1 | 0.407696 | 0.036148 | 0.627725 | 0.4467 | 0.144646 | 0.439369 |
| WD | 0.183851 | 1 | 0.13459 | 0.686255 | 0.374659 | 0.321199 | 0.555837 |
| RTD | 0.110513 | 0.174914 | 1 | 0.42689 | -0.06396 | 0.88564 | 0.327935 |
| LDMC | 0.705812 | 0.448198 | 0.513225 | 1 | 0.35851 | 0.509183 | 0.808581 |
| WDMC | 0.534603 | 0.473692 | 0.098398 | 0.632114 | 1 | 0.057385 | 0.195057 |
| RDMC | 0.375654 | 0.291657 | 0.811825 | 0.641761 | 0.40463 | 1 | 0.46465 |
| LTD | 0.609707 | 0.330367 | 0.488053 | 0.878782 | 0.538674 | 0.569289 | 1 |

**Table S6**. Number of field sites where relative growth rates of each species were measured on wild-grown mature trees and wild-grown juveniles.

| **Species** | **Mature trees**  **(# sites/30)** | **Wild-grown juveniles**  **(# sites/3)** |
| --- | --- | --- |
| *Agathis_australis* | 2 | 1 |
| *Alectryon_excelsus* | 2 | 0 |
| *Aristotelia_serrata* | 7 | 0 |
| *Beilschmiedia_tarairi* | 1 | 1 |
| *Beilschmiedia_tawa* | 9 | 2 |
| *Carpodetus_serratus* | 20 | 1 |
| *Coprosma_arborea* | 1 | 1 |
| *Coprosma_linariifolia* | 10 | 0 |
| *Corynocarpus_laevigatus* | 1 | 1 |
| *Dacrydium_cupressinum* | 15 | 3 |
| *Dacrycarpus_dacrydioides* | 6 | 2 |
| *Dracophyllum_longifolium* | 9 | 0 |
| *Dysoxylum_spectabile* | 5 | 1 |
| *Elaeocarpus_dentatus* | 9 | 2 |
| *Elaeocarpus_hookerianus* | 15 | 1 |
| *Fuchsia_excorticata* | 8 | 0 |
| *Griselinia_littoralis* | 23 | 0 |
| *Halocarpus_biformis* | 5 | 0 |
| *Hedycarya_arborea* | 11 | 1 |
| *Hoheria_populnea* | 1 | 1 |
| *Ixerba_brexioides* | 3 | 1 |
| *Knightia_excelsa* | 9 | 2 |
| *Kunzea_ericoides* | 7 | 0 |
| *Laurelia_novae-zelandiae* | 7 | 2 |
| *Leptospermum_scoparium* | 6 | 0 |
| *Litsea_calicaris* | 4 | 1 |
| *Melicytus_ramiflorus* | 16 | 1 |
| *Metrosideros_robusta* | 5 | 0 |
| *Metrosideros_umbellata* | 10 | 0 |
| *Myrsine_australis* | 18 | 1 |
| *Myrsine_divaricata* | 20 | 0 |
| *Myrsine_salicina* | 6 | 1 |
| *Nestegis_lanceolata* | 2 | 1 |
| *Fuscospora_cliffortioides* | 0 | 1 |
| *Fuscospora_fusca* | 12 | 0 |
| *Lophozonia_menziesii* | 14 | 0 |
| *Fuscospora_solandri* | 15 | 0 |
| *Fuscospora_truncata* | 3 | 0 |
| *Olearia_rani* | 8 | 1 |
| *Pennantia_corymbosa* | 7 | 0 |
| *Phyllocladus_alpinus* | 16 | 0 |
| *Phyllocladus_trichomanoides* | 4 | 1 |
| *Pittosporum_eugenioides* | 8 | 0 |
| *Pittosporum_tenuifolium* | 7 | 0 |
| *Podocarpus_hallii* | 22 | 2 |
| *Podocarpus_totara* | 3 | 1 |
| *Prumnopitys_ferruginea* | 17 | 3 |
| *Prumnopitys_taxifolia* | 5 | 1 |
| *Pseudopanax_arboreus* | 1 | 0 |
| *Pseudowintera_colorata* | 17 | 0 |
| *Pseudopanax_crassifolius* | 23 | 1 |
| *Quintinia_serrata* | 0 | 0 |
| *Schefflera_digitata* | 5 | 1 |
| *Sophora_microphylla* | 9 | 0 |
| *Weinmannia_racemosa* | 17 | 1 |
| *Weinmannia_silvicola* | 2 | 1 |

**Table S7.** Results of the meta-analysis of individual site-level correlations between traits and relative growth rates measured on wild juvenile trees (*N* = 3 sites).

| **Site** | **LMA-RGR_95_ correlations** | **WD-RGR_95_ correlations** | **RTD-RGR_95_ correlations** | **# species (N)** |
| --- | --- | --- | --- | --- |
| 1 | -0.3186 | -0.2281 | -0.4024 | 11 |
| 2 | -0.1418 | -0.5906 | 0.5243 | 6 |
| 3 | -0.1439 | -0.137 | -0.1261 | 25 |
|  |  |  |  |  |
| **Random effects model results** | |  |  |  |
| Correlation | -0.1874 | -0.2069 | -0.1277 |  |
| *z* | -1.0893 | -1.2057 | -0.6506 |  |
| *P*-value | 0.276 | 0.2279 | 0.5153 |  |
|  |  |  |  |  |
|  |  |  |  |  |
| **Test of Heterogeneity** | |  |  |  |
| *Tau*^2^ | 0 | 0 | 0.0177 |  |
| *I*^2^ | 0% | 0% | 12.5 |  |
| *Q* | 0.21 | 0.78 | 2.29 |  |
| df | 2 | 2 | 2 |  |
| *P*-value | 0.9011 | 0.6781 | 0.319 |  |

**Table S8.** Results of the meta-analysis of individual site-level correlations between traits and relative growth rates measured on wild mature trees (*N* = 30 sites).

| **Site** | **LMA-RGR_95_ correlations** | **WD-RGR_95_ correlations** | **RTD-RGR_95_ correlations** | **# species (N)** |
| --- | --- | --- | --- | --- |
| 1 | -0.813895237 | 0.462610183 | -0.165376601 | 8 |
| 2 | 0.381622962 | -0.314764815 | 0.650247638 | 6 |
| 3 | -0.426153112 | 0.16758065 | -0.131730679 | 10 |
| 4 | 0.124034009 | 0.128390639 | -0.244841292 | 17 |
| 5 | 0.738939364 | -0.984252137 | 0.608800252 | 3 |
| 6 | 0.078420164 | 0.184561918 | -0.140038208 | 22 |
| 7 | -0.27617209 | 0.101742213 | 0.384251951 | 14 |
| 8 | -0.252016209 | -0.452348006 | 0.2478125 | 12 |
| 9 | -0.200117262 | 0.657333651 | -0.02664192 | 17 |
| 10 | 0.022084763 | -0.319186698 | -0.186020623 | 13 |
| 11 | -0.355738019 | 0.018284221 | 0.042198785 | 17 |
| 12 | -0.547235871 | 0.10755168 | 0.312858831 | 15 |
| 13 | -0.384428062 | -0.151569639 | -0.067079108 | 18 |
| 14 | -0.039036739 | -0.042780669 | 0.350134387 | 27 |
| 15 | 0.336247674 | -0.199763977 | -0.07686687 | 14 |
| 16 | 0.319912594 | -0.314835302 | -0.429018735 | 14 |
| 17 | -0.537107977 | 0.102606374 | 0.105983262 | 16 |
| 18 | -0.116327929 | 0.330439618 | 0.049938176 | 13 |
| 19 | 0.056637452 | -0.201939117 | -0.209981799 | 31 |
| 20 | 0.216216966 | -0.151712502 | 0.01660467 | 25 |
| 21 | 0.625711606 | -0.180599102 | -0.096659951 | 11 |
| 22 | -0.042385775 | -0.21397391 | 0.067913332 | 24 |
| 23 | -0.144944861 | -0.730023948 | 0.979073405 | 5 |
| 24 | -0.246579468 | -0.26367079 | 0.269639761 | 12 |
| 25 | -0.149511643 | -0.511503602 | -0.113554954 | 32 |
| 26 | -0.109932611 | -0.072042481 | 0.681906606 | 11 |
| 27 | -0.111100179 | -0.484262901 | -0.157909591 | 15 |
| 28 | 0.01658383 | -0.016027817 | 0.189454696 | 26 |
| 29 | 0.145023599 | 0.28300185 | -0.162025222 | 16 |
| 30 | -0.09873755 | -0.191897401 | 0.162590523 | 22 |
|  |  |  |  |  |
| **Random effects model results** | |  |  |  |
| Correlation | -0.0805 | -0.0773 | 0.0442 |  |
| *z* | -1.5023 | -1.3688 | 0.8123 |  |
| *P*-value | 0.133 | 0.171 | 0.4166 |  |
|  |  |  |  |  |
| **Test of Heterogeneity** | |  |  |  |
| Tau^2^ | 0.0085 | 0.0163 | 0.0104 |  |
| *I*^2^ | 10% | 17.6% | 12% |  |
| *Q* | 32.23 | 35.19 | 32.95 |  |
| df | 29 | 29 | 29 |  |
| *P*-value | 0.3097 | 0.1985 | 0.28 |  |

**References**

**Kramer-Walter KR, Bellingham PJ, Millar TR, Smissen RD, Richardson SJ, Laughlin DC. 2016.** Root traits are multidimensional: specific root length is independent from root tissue density and the plant economic spectrum. *Journal of Ecology* **104**: 1299-1310.
